# The changing landscape of *Plasmodium falciparum* drug resistance in the Democratic Republic of Congo

**DOI:** 10.1186/s12879-019-4523-0

**Published:** 2019-10-22

**Authors:** Molly Deutsch-Feldman, Ozkan Aydemir, Margaret Carrel, Nicholas F. Brazeau, Samir Bhatt, Jeffrey A. Bailey, Melchior Kashamuka, Antoinette K. Tshefu, Steve M. Taylor, Jonathan J. Juliano, Steven R. Meshnick, Robert Verity

**Affiliations:** 1 Department of Epidemiology, Gillings School of Global Public Health, University of North Carolina, Chapel Hill, USA; 2 Department of Pathology and Laboratory Medicine, Brown University, Providence, RI USA; 3 Department of Geographical & Sustainability Sciences, University of Iowa, Iowa City, IA USA; 4 Medical Research Council Centre for Global Infectious Disease Analysis, Department of Infectious Disease Epidemiology, Imperial College London, London, UK; 5 Ecole de Santé Publique, , Faculté de Médecine, University of Kinshasa, Kinshasa, Democratic Republic of Congo; 6 Division of Infectious Diseases and Duke Global Health Institute, Duke University, Durham, NC USA; 7 Division of Infectious Diseases, University of North Carolina at Chapel Hill, Chapel Hill, USA; 8 Curriculum in Genetics and Molecular Biology, University of North Carolina at Chapel Hill, Chapel Hill, USA

**Keywords:** Malaria, Drug resistance, Spatial-temporal modeling

## Abstract

**Background:**

Drug resistant malaria is a growing concern in the Democratic Republic of the Congo (DRC), where previous studies indicate that parasites resistant to sulfadoxine/pyrimethamine or chloroquine are spatially clustered. This study explores longitudinal changes in spatial patterns to understand how resistant malaria may be spreading within the DRC, using samples from nation-wide population-representative surveys.

**Methods:**

We selected 552 children with PCR-detectable *Plasmodium falciparum* infection and identified known variants in the *pfdhps* and *pfcrt* genes associated with resistance. We compared the proportion of mutant parasites in 2013 to those previously reported from adults in 2007, and identified risk factors for carrying a resistant allele using multivariate mixed-effects modeling. Finally, we fit a spatial-temporal model to the observed data, providing smooth allele frequency estimates over space and time.

**Results:**

The proportion of co-occurring *pfdhps* K540E/A581G mutations increased by 16% between 2007 and 2013. The spatial-temporal model suggests that the spatial range of the *pfdhps* double mutants expanded over time, while the prevalence and range of *pfcrt* mutations remained steady.

**Conclusions:**

This study uses population-representative samples to describe the changing landscape of SP resistance within the DRC, and the persistence of chloroquine resistance. Vigilant molecular surveillance is critical for controlling the spread of resistance.

## Background

With 17 million confirmed cases in 2016, the burden of malaria in the Democratic Republic of the Congo is one of the highest in the world [1]. Understanding malaria dynamics in DRC is critical in eliminating malaria from sub-Saharan Africa. Previous studies of *P. falciparum* genetic diversity within the DRC have shown a mixture of both West and East African strains, indicating that the DRC serves as a nexus of regional transmission, incorporating parasites from both sides of the continent [2–4]. Insights from genetic *P. falciparum* studies within the DRC therefore have important implications for reducing disease burden within the country and for Central and sub-Saharan Africa.

Efforts to halt transmission in the DRC, and across Africa, are being threatened by growing resistance to commonly used antimalarial drugs [5, 6]. Molecular markers can be used to identify resistant infections to monitor the spread of resistance [7, 8]. These markers include mutations in the dihydropteroate synthase (*pfdhps*) gene, which, along with mutations of the dihydrofolate reductase (*pfdhfr*) gene, confer resistance to sulfadoxine [7–9]. Specifically, the *pfdhps* A437G, K540E, and A581G mutations are associated with sulfadoxine/pyrimethamine (SP) treatment failure [8, 9]. Co-occurrence of the K540E and A581G mutations has been associated with failure of SP for intermittent preventive therapy during pregnancy (IPTp), a program recommended for all pregnant women in the DRC [10, 11]. Additionally, mutations of the chloroquine resistance transporter (*pfcrt*) gene, specifically mutations in amino acids 72–76 (wild type CVMNK), confer chloroquine resistance [12, 13]. Past work has demonstrated that the K76 T mutation alone increases the risk of chloroquine treatment failure, though a K76 T-containing CVIET triple mutant haplotype has emerged in many African countries, including the DRC [5, 7, 14]. Studies have demonstrated that this haplotype is also associated with amodiaquine treatment failure; amodiaquine is used as part of the first line therapy for malaria in the DRC [10, 15–17]. Additionally, another *pfcrt* haplotype, SVMNT, is also associated with resistance to amodiaquine [12, 17, 18]. Monitoring these molecular markers is critical for halting the spread of resistance.

Studies conducted in the DRC have demonstrated spatial structure of parasites resistant to SP and chloroquine [5, 6, 19]. Amongst adult respondents to the 2007 Demographic and Health Survey (DHS), those infected with parasites with a single *pfdhps* A437G mutation were spread throughout the country, though mostly located in the western part of the DRC [6]. Co-occurring K540E and A581G mutants were rarer, but showed geographic clustering in the northeast region of the country [6]. Past studies from the DRC have demonstrated that the *pfdhfr* mutations are nearly fixed within the population [20]. There was no apparent clustering of the *pfcrt* CVIET haplotype [21]. More recent work demonstrated similar patterns for *pfdhps* mutations amongst children sampled in 2013 [19]. However, the *pfcrt* CVIET haplotype displayed a pattern of concentrated cases on the eastern and western borders, with fewer mutations observed in the center of the DRC [19]. These findings highlight hotspots of resistance within the DRC.

Previous geospatial studies of drug resistance in the DRC often do not use population based samples and are therefore not necessarily nationally representative [22]. Better estimates of the burden and distribution of these mutations can be obtained using nationally representative surveys [19, 23]. This is the first study, to our knowledge, that uses data from a nationally representative database to evaluate risk factors for carrying a resistant infection and to study longitudinal changes in resistance.

Here, we describe the changing spatial patterns of SP and chloroquine resistance over time in the DRC by comparing samples from the 2013–2014 DHS to previously published data drawn from the 2007 DHS. We begin by describing the proportion of single and co-occurring mutations in both 2007 and in 2013. Next, we present an epidemiologic risk factor analysis to identify covariates associated with increased prevalence of resistant infections. The findings from this analysis will help identify individuals and communities that may be of higher risk for resistant infections. Finally, we use Markov chain Monte Carlo to fit a spatial-temporal model to the observed data to explore whether the geographic range of drug resistant mutations has shifted between 2007 and 2013. This model allows us to directly compare allele frequencies across space and time.

## Methods

### Study population

Samples were drawn from the DHS survey, conducted in the DRC in 2013–2014 [24, 25]. The DHS Program conducts cross-sectional, nationally representative population health surveys in over 90 countries. In the DRC, the DHS survey uses a randomized cluster sampling method [26]. For the 2013–2014 survey, 536 geographic clusters across the DRC were randomly selected. Next, households were randomly selected from these clusters for inclusion in the DHS. The 2013–2014 survey included adolescents and adults ages 15–59 and children under age 5. DHS survey conductors visited selected households and obtained informed consent from each individual age 18 or older, or from a parent or legal guardian for children and adolescents under age 18. Survey conductors administered an extensive questionnaire covering topics such as socioeconomic status, education, and health history. Each individual was administered a malaria rapid diagnostic test and blood samples were collected on filter paper and shipped to the University of North Carolina for molecular diagnostic testing. All DHS questionnaires and procedures have been approved by the ICF Institutional Review Board and comply with the United States Department of Health and Human Services regulations for the protection of human subjects. This study was approved by the Internal Review Board at The University of North Carolina, Chapel Hill and at the Kinshasa School of Public Health.

A previous DHS survey was conducted in the DRC in 2007 [24]. Similar to 2013–2014, a two-stage random cluster sampling scheme was used to select households for inclusion, though only adults were asked to participate. The 2007 survey used 300 sampling clusters (fewer than in 2013–2014) and the clusters were not the same between years.

### DNA amplification and genotyping

Findings from the 2007 DHS have been previously published [3–6, 27]. As described, 220 samples were previously genotyped at the *pfdhps* and *pfcrt* loci [6, 28].

Molecular diagnostic testing for malaria parasites was completed for all individuals included in the 2013–2014 DHS [23, 29]. Unlike children included in previous studies, these children participated in the DHS and thus have extensive individual demographic data [19]. DNA was extracted from filter paper using a Chelex-100 Kit (Bio-Rad, Richmond, CA). Samples were tested in duplicate using a real-time PCR assay to target the *P.f. lactate dehydrogenase* gene; human *beta-tubulin* was used as a positive control. Primer sequences for both genes have been previously published [18, 30]. Samples in which both replicates amplified parasite DNA were considered positive. If one replicate failed to amplify but the other amplified with a PCR cycle threshold (C_T_) value below 38 that sample was also considered positive [23].

A total of 552 children with PCR-confirmed *P.falciparum* infections from 536 clusters were selected from the 2013–2014 DHS for inclusion in this study. Overall, 7137 children included in the DHS had complete data; malaria prevalence by PCR was 38.6% [23]. Children with C_T_ values under 30 were chosen for this analysis to ensure sufficient sequencing coverage. Children were selected from throughout the DRC, providing ample geographic representation. Samples from the selected children were amplified using molecular inversion probes (MIPs), a multistep protocol that allows for highly multiplexed deep sequencing [19]. MIPs were designed to flank the *pfdhps* and *pfcrt* targets. Each sample was individually barcoded in order to de-multiplex sequences and yield individual level data. Sequencing data was processed using the MIPWrangler software, as previously described [19]. Paired-end reads were stitched and filtered by base quality scores, expected length, a minimum unique molecular index (UMI) count of 3 and minimum relative abundance of 0.5% within sample. SNP calls were further filtered to have a minimum Phred quality score of 20. Mixed infections were identified as those with heterozygous SNP calls at any of the genotyped loci.

### Comparison of allele frequencies

Proportions of each SNP were calculated for each year and compared using the UpSet package in the R statistical language [31, 32]. Mixed infections with both referent and mutant genotype calls were considered mutant. The UpSet package does not accommodate missing data; therefore, this analysis only included observations with SNP calls at all sites. Frequencies were statistically compared between years using chi-squared tests. For these tests, individuals missing a genotype call at any given site were not included in the analysis for that site only. COI estimates were determined using THE REAL McCOIL software [33].

### Epidemiologic risk factor analysis

Risk factor data was drawn from all surveyed individuals within a cluster. Potential cluster and individual level demographic risk factors were based on biological plausibility and by consulting relevant literature [27, 34]. Both cluster-level and individual-level risk factors were evaluated as several studies of malaria conducted in the DRC have demonstrated the role of community level factors on individual infection risk [27, 34, 35]. Selected cluster-level covariates included: malaria prevalence by PCR, percentage of individuals in the lowest wealth category, percentage of individuals without education, percentage of pregnant women who took SP, percentage of children who took chloroquine for a fever or cough, cluster size, and urban vs rural status. Individual covariates included wealth index and biological sex. Age could not be included as the DHS does not collect data from individuals aged 5–15, thus there is a gap in the age distribution.

Multivariate mixed-effect Poisson models were used to identify associations between selected covariates and the probability of having a drug resistant infection. Using a Poisson distribution and estimating a robust variance is an alternative to fitting log-risk models (that use a binomial distribution), which often do not converge [36, 37]. Full specification of the model is available in Additional file 1: Text S1. We used backwards selection to identify significant associations, initially fitting a full model with all previously mentioned potential risk factors. Covariates were subsequently removed one at a time based on the highest *p*-value until only covariates with *p*-values less than or equal to 0.05 were left. Secondary analyses were conducted using univariate models for each potential risk factor to determine if the marginal associations from the univariate models matched those of the multivariate model. To account for dependency between individuals living in the same province, all models fit random effects for DHS province; DHS cluster could not be used as there were too few observations per cluster to estimate random effects. All analyses were conducted in the R statistical language using the lme4 package [38].

### Spatial prediction models

Spatial prediction maps were generated by fitting a spatial-temporal model to the data. This model assumed a smooth surface based on the logistic Gaussian process [39] to describe the underlying frequency of resistant alleles as a function of space, time, and a number of covariates. Observed counts of resistant alleles were modelled as binomial draws from the underlying frequency distribution. The complete model specification can be found in Additional file 1: Text S1. Covariates used in the model included accessibility, night time lights (a measure of population density), and proportion urban/rural [40–42]. Every covariate was given a weighting parameter allowing it to have a greater or lesser effect on the data, and these parameters were given suitable priors. To facilitate model fitting and to ensure our method scaled well with the number of dimensions and DHS clusters, the full model was approximated using 250 random Fourier features (Additional file 1: Text S1) [43]. Model fitting was conducted via Hamiltonian Monte Carlo (HMC) using the GRETA package [44] in the R statistical language. In each analysis the HMC was run for 10,000 burn-in iterations and 1 million sampling iterations, thinning to every 100th sample to remove autocorrelation. Posterior parameter values were sampled at random to generate 1000 maps for each of the *pfdhps* mutations A437G, K540E, A581G, and the *pfcrt* CVIET haplotype, and these 1000 maps were summarized in the form of a mean prediction map and standard deviation (error) map.

## Results

### MIP analyses of 2013–2014 samples

**Fig. 1:**
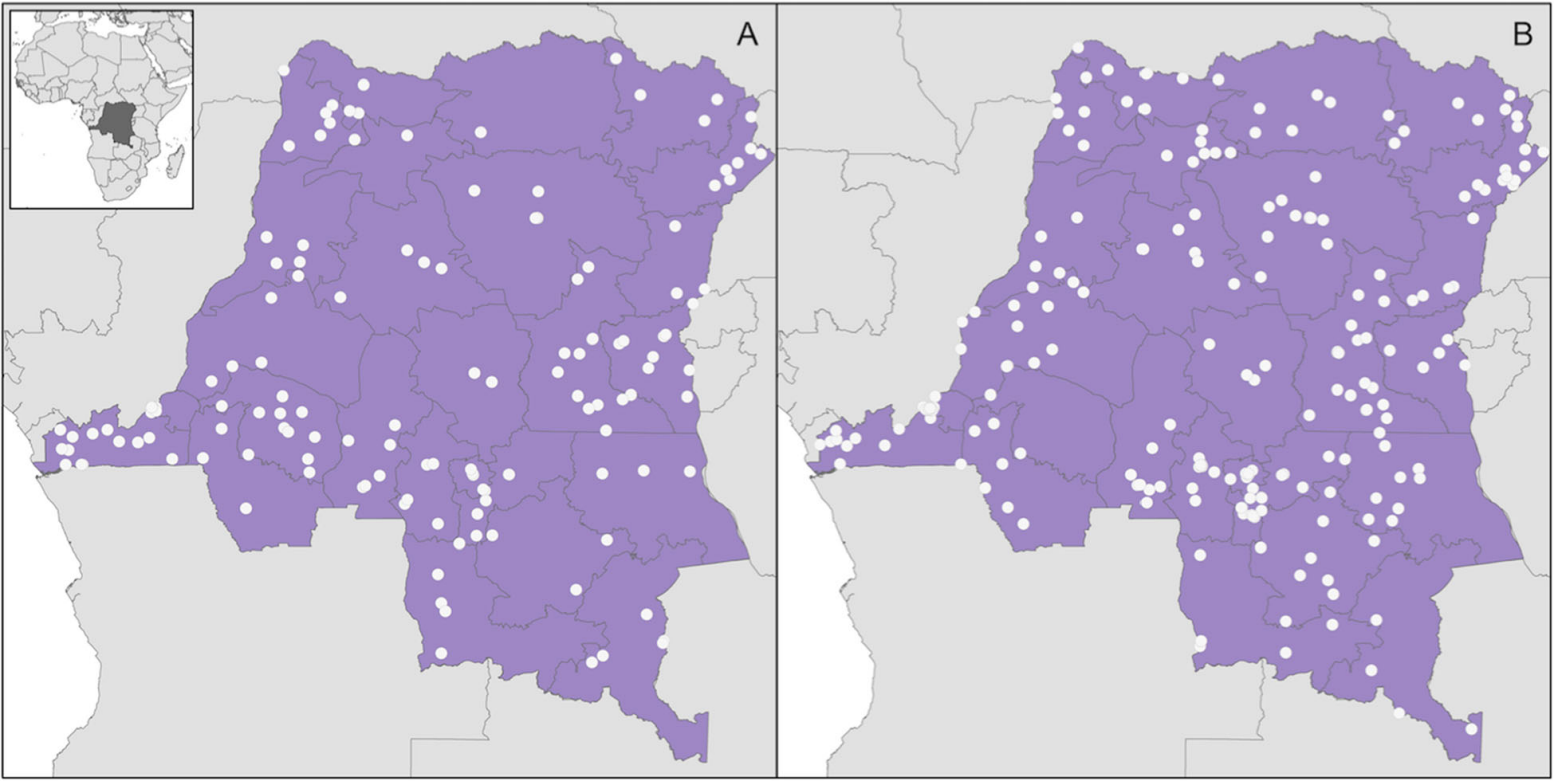
DHS cluster locations of the children included in the analysis. Clusters are from 2007 (**a**) and 2013 (**b**). The 26 DRC municipal province borders are outlined in black

Following MIPWrangler processing, a 250 bp paired end MiSeq run following a single MIP capture yielded 9 million paired end reads and 4 million UMIs. Sequencing was successful for 514/552 children. The geolocation data indicates that these 514 children live throughout the DRC (Fig. 1). Complete *pfcrt* SNP data was available for 513 children, and 307 had data available across all *pfcrt* and *pfdhps* loci of interest.

The results of THE REAL McCOIL analysis estimated an average complexity of infection (COI) of 2 (range = 1–17). Of children with complete genotyping data, 108 (35% of the total) had polyclonal infections, compared with 20% of infections that were polyclonal in 2007 (X^2^ = 7.28, df = 1, *p* <  0.01). However, this is likely an underestimate of the true number of polyclonal infections as we are only looking at three loci.

### Frequency of *pfdhps* and *pfcrt* variants over time

**Fig. 2:**
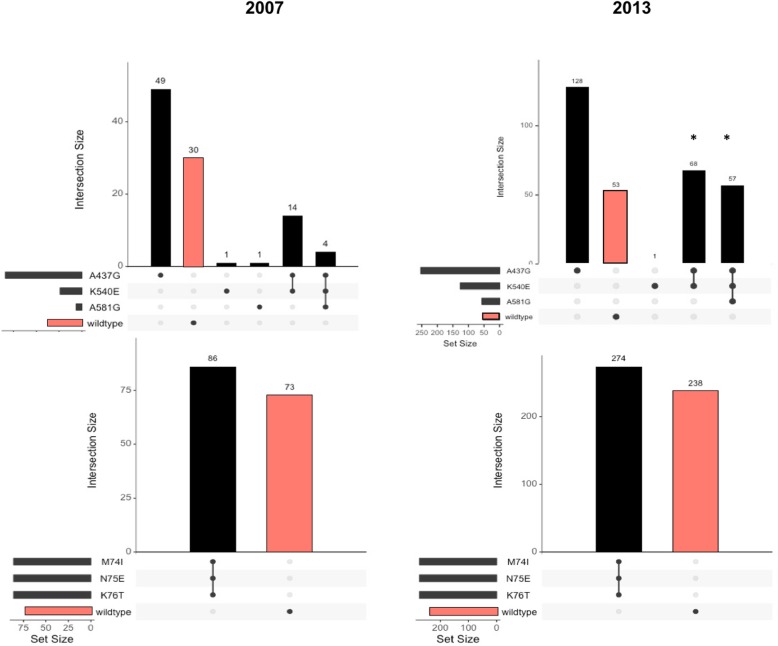
Frequencies of *pfdhps* and *pfcrt* mutations in 2007 and 2013. Wild-type genotypes are highlighted in red. Chi-squared tests were performed to statistically compare proportions; asterisks indicate a statistically significant difference in proportion between years

The overall proportion of *pfdhps* mutations remained relatively steady from 2007 to 2013, (80% [95% CI = 72–86%] vs 86% [95% CI = 83–89%], Fig. 2). However, the proportions of K540E mutations increased significantly from 17% (95% CI = 11–24%) in 2007 to 41% (95% CI = 36–47%) in 2013 (X^2^ = 25.57, df = 1, *p* <  0.01). A581G mutations also increased significantly between years, from 3% (95% CI = 1–8%) in 2007 to 18% (95% CI = 14–23%) in 2013 (X^2^ = 15.27, df = 1, *p* < 0.01). Only one individual in 2007 had a single A581G mutation, in all other cases, in both years, A581G was only found in the presence of a K540E mutation. Thus, the proportion of double K540E/A581G mutants also increased significantly across years, from 2% (95% CI = 1–7%) in 2007 to 18% (95% CI = 14–23%) in 2013 (X^2^ = 19.27, df = 1, *p* < 0.001).

Amongst monoclonal infections, there were similar patterns of allele frequencies over time. The proportions of infections carrying any of the three *pfdhps* SNPs increased slightly; 62% (95% CI = 51–73%) in 2007 versus 73% (95% CI = 66–79%) in 2013 (X^2^ = 2.71, df = 1, *p* = 0.10). However, the proportion of double K540E and A581G mutant parasites increased from 4% (95% CI = 1–8%) in 2007 to 12% (95% CI = 7–17%) in 2013 (X^2^ = 3.03, df = 1, *p* = 0.08).

The proportion of *pfcrt* CVIET haplotypes did not change significantly from 2007 (58% [95% CI = 50–65%] to 2013 (54% [95% CI = 49–58%]; X^2^ = 0.80, df = 1, *p* = 0.37). No parasites harbored the SVMNT haplotype. Among monoclonal infections, the proportion of *pfcrt* CVIET haplotypes also remained steady; 55% (95% CI = 46–63%) in 2007 and 56% (95% CI = 51–61%) in 2013 (X^2^ = 0.012, df = 1, *p* = 0.91).

### Risk factor analysis

Complete *pfdhps* and DHS covariate data were available for 492 individuals from both the 2007 and 2013–2014 studies; complete *pfcrt* and DHS covariate data was available for 675 individuals. Reported antimalarial use was low, with a cluster average of only 12% of pregnant women receiving SP in 2007 and 24% in 2013. In 2007, an average of only 4% of children per cluster reporting a cough or fever received amodiaquine, and only about 1% in 2013. A summary of the cluster and individual level characteristics by *pfdhps* and *pfcrt* genotype is available in Table 1.

**Table 1:** Individual and cluster level characteristics of all study participants, stratified by *Pfdhps* and *Pfcrt* genotype

	*Pfdhps*			*Pfcrt*		
	Wildtype (*N* = 81)	Any *pfdhps* mutation (*N* = 434)	*P*-value^***^	Wildtype (*N* = 306)	CVIET haplotype (*N* = 369)	*P*-value^***^
Malaria prevalence (SD)	59.3 (20.4)	58.9 (21.8)	0.872	60.04 (21.74)	57.42 (22.24)	0.125
Anti-malarial use during pregnancy^a^ (SD)	16.7 (16.6)	22.2 (18.1)	0.011	2.0 (6.2)	1.9 (6.0)	0.955
Anti-malarial use amongst children (SD)^b^	3.0 (6.4)	2.0 (5.6)	0.126	1.7 (4.4)	1.6 (4.0)	0.745
Mean DHS Cluster size (SD)	17.9 (18.0)	19.3 (18.8)	0.652	17.8 (19.07)	20.0 (22.14)	0.266
% without education (SD)	32.7 (23.48)	23.1 (21.5)	< 0.001	28.8 (24.41)	22.0 (20.16)	< 0.001
% in lowest wealth category (SD)	30.2 (23.0)	21.4 (22.5)	0.001	27.2 (22.5)	20.5 (22.5)	< 0.001
Number living in urban area (%)	28 (34.6)	154 (35.4)	0.975	90 (29.4)	135 (36.6)	0.059
Individual covariates:						
Number female (%)	41 (50.6)	228 (52.5)	0.845	153 (50.0)	192 (52.0)	0.654
Median Individual Wealth Index (IQR)	2 (1–3)	3 (1–4)	0.015	2 (1–3)	3 (2–4)	< 0.001

*** *p*-values for tests conducted for comparisons between wildtype and mutant groups (Chi-squared tests for categorical data and t-tests for continuous data)

^a^ Percentage of pregnant women reporting drug use; SP use is described by *pfdhps* status and chloroquine use by *pfcrt* status

^b^ Percentage of children with a cough or fever that received SP or chloroquine; SP use is described by *pfdhps* status and chloroquine use by *pfcrt* status

The mixed-effects model identified several risk factors for *pfdhps* mutations and the *pfcrt* CVIET haplotype (Table 2). Increasing cluster-level use of SP was a risk factor for carrying a K540E mutation (PR = 1.14, 95% CI = 1.09–1.20, *p* < 0.01) as was increasing cluster prevalence of *P. falciparum* infections (PR = 1.11, 95% CI = 1.06–1.17, *p* = 0.02). The results from the *pfcrt* model indicated an inverse relationship between the prevalence of mutations and the proportion of uneducated individuals (PR = 0.92, 95% CI = 0.90–0.95, *p* < 0.01). Education may be a proxy for access to medications.

**Table 2:** Risk factors identified from final backwards selection multivariate risk factor model

Covariate	Prevalence Ratio (95% CI)	*P*-value
*Pfdhps K540E*		
10% increase in malaria prevalence	1.11 (1.06–1.17)	0.024
10% increase in cluster SP use^a^	1.14 (1.09–1.20)	< 0.01
*Pfcrt CVIET*		
10% increase in lowest education category	0.92 (0.90–0.95)	< 0.01

^a^ reported SP use amongst pregnant women

Increasing cluster level SP use amongst pregnant women and malaria prevalence were both identified as risk factors for carrying the K540E mutation (including those with the A581G mutation also), while education was the only risk factor identified for carrying the CVIET haplotype.

Results from the secondary univariate models matched those from the multivariate models (Additional file 1: Table S2). Like the multivariate model, the univariate models did not identify any risk factors for carrying any *pfdhps* mutation. The univariate models of K540E identified both increasing SP use and increasing cluster *P.f.* prevalence as risk factors, though the *p*-value for prevalence was not significant at the 5% level. Like the multivariate model, the univariate models of *pfcrt* identified only increasing cluster level education as a risk factor for the CVIET haplotype. Similarly, increasing cluster level proportion of poor individuals showed a protective effect against the CVIET haplotype, though the association had a *p*-value that was not significant at the 5% level. Full results for the univariate models are available in Additional file 1: Table S1.

### Spatial-temporal prediction maps

**Fig. 3:**
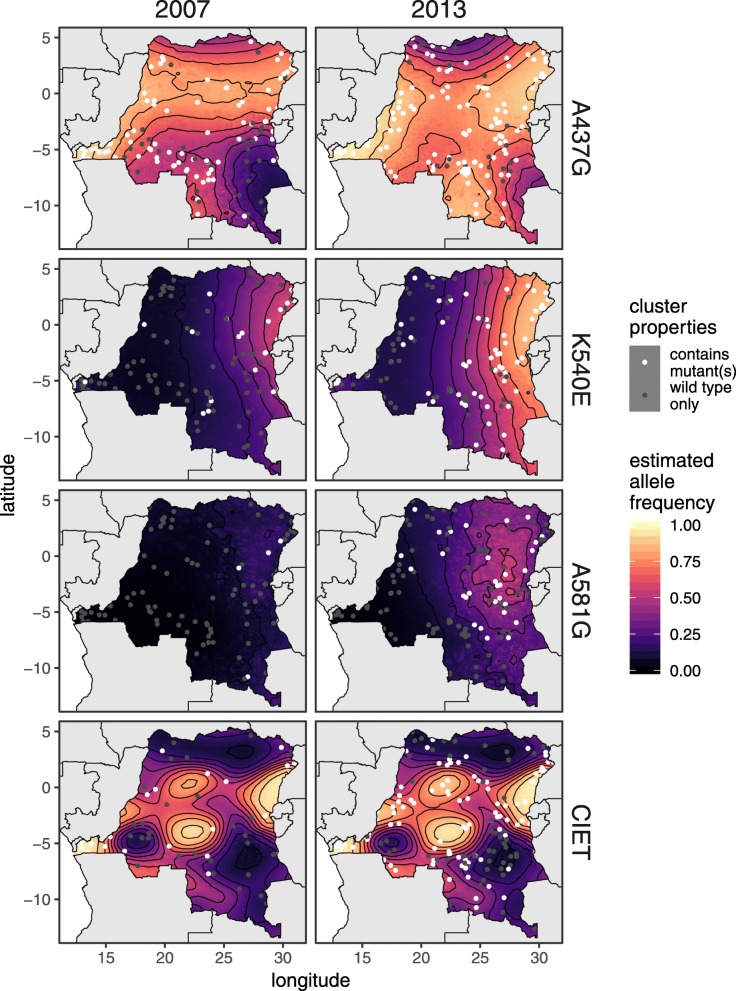
Spatial prediction maps comparing prevalence and spatial distribution of *pfdhps* and *pfcrt* mutations. Predictions were generated for 2007 (left panels) and 2013 (right panels). Clusters with at least one mutation are marked with a white “x”, clusters with no mutations are marked in grey circles. Horizontal black lines represent a 10% increase in prevalence

The prediction maps generated from the logistic Gaussian model indicate that the allele frequency distribution of the A437G mutation shifted range slightly between 2007 and 2013, decreasing in the east and west of the country but increasing in the south (Fig. 3). The results also demonstrate the geographic spread of both the K540E and A581G mutations from east to west, showing both an increase in the frequency of each mutation and a geographic expansion, indicated by the shift in the 10% contour lines (marked in black). *Pfcrt* results demonstrate that there has been no significant change in the spatial distribution of the CVIET haplotype between 2007 and 2013; the prevalence of the haplotype is highest across the central part of DRC. The wide 95% credible intervals on posterior parameter weights indicate that there is large uncertainty as to which components are driving the signal (Additional file 1: Figure S1). Similarly, the posterior error maps show that there is large uncertainty in the predicted allele frequency at most points in space (Additional file 1: Figure S2). Hence, it is important to recognize that the maps in Fig. 3 show only the average prediction, and there are alternative maps that are plausible under the posterior distribution. However the general patterns described above, such as the east-west expansion of K540E and A581G mutations, remain consistent over the majority of posterior draws, and therefore are well-supported in spite of uncertainty in any specific prediction.

## Discussion

Monitoring the changing spatial distribution of drug resistance markers is necessary for developing efficient interventions to halt the spread of resistance and eliminate malaria. Here, we leverage geolocated samples from the DHS to measure resistance mutations across the DRC and map changes that occurred between 2007 and 2013 [6, 19, 21]. Studies using nationally representative samples like the DHS are less susceptible to selection bias; however, longitudinal comparisons of DHS surveys have been impeded by the fact that the individual survey clusters change between surveys. Here, we use a spatial prediction model that overcomes this by assuming a continuous surface of underlying allele frequencies, allowing us to integrate information at different points in space and time.

This study found that the 540 and 581 *pfdhps* mutations have increased in the DRC since 2007, both in numbers and in geographic spread. This agrees with recent findings of an increase in *pfdhps* mutations between 2014 and 2015 amongst individuals living in southwest DRC [45]. Evidence of geographic expansion from the eastern part of DRC is also supported by previous research that demonstrated higher prevalence of both mutations in East Africa compared to West Africa [9, 46]. This expansion is particularly concerning as these mutations are associated with SP failure during IPTp [11, 19, 46]. The risk factor analysis indicates that these increases may be in part driven by SP use, which was associated with increased prevalence of *pfdhps* mutations. Further, this study indicates that increasing community level drug use, not necessarily individual use, is associated with increases in resistance. This is consistent with previous work that demonstrated associations between community level interventions and malaria risk [34, 35].

Chloroquine resistance has remained relatively steady since 2007; the proportion of CVIET parasites is unchanged and the spatial distribution remains similar. These findings are troubling as the DRC halted chloroquine use as a first line treatment in 2001 due to concerns about growing resistance [47, 48]. This sustained resistance may be in part driven by demographic factors; the risk factor models results indicate that cluster-level education and wealth are associated with chloroquine resistance. There may also be unregulated chloroquine use, as has been reported in other sub-Saharan African countries [49]. Additionally, there is evidence that the CVIET haplotype is associated with amodiaquine resistance [15, 16, 50]. Since amodiaquine is used as part of the first line treatment ASAQ in the DRC, this association may explain why the prevalence of CVIET has remained steady over time [16, 48, 50]. Reported ASAQ use was too low in this study for us to evaluate this relationship statistically. However, we did not detect the SVMNT haplotype, also found to be associated with ASAQ resistance, in this population [17, 18].

The findings from this study have direct implications for malaria control programs in the DRC. As mentioned, SP is still used in the DRC as the primary drug for IPTp [47, 48]. Increasing SP resistance may threaten these preventive efforts. Additionally, though chloroquine is no longer a recommended treatment for malaria, reports from other sub-Saharan African countries show a steep drop in the proportion of resistant parasites after ending chloroquine use [48, 51, 52]. The sustained prevalence of chloroquine resistance seen in this study is alarming and warrants further investigation.

Effective monitoring of drug resistance requires sensitive molecular tools that can accommodate a large number of samples. Using MIPs to amplify resistance loci allows for highly multiplexed and efficient deep sequencing of *Plasmodia*. This study demonstrates the utility of MIPs for drug resistance surveillance, and the ability to answer critical epidemiological questions. This novel method can also be used to investigate questions of parasite population structure, gene flow, and selective sweeps, amongst others. The spatial-temporal approach used here also represents a step forward compared with previous mapping efforts [19]. The random Fourier features (RFF) method allows us to explore complex models in a computationally efficient way, thereby reducing the time and resources required to perform this kind of advanced spatial analysis and opening the door to much larger datasets in the future.

There are several limitations to this study. First, we only have access to a relatively small number of samples distributed over a wide geographic area, and this is reflected in the large credible intervals around our spatial-temporal predictions. We can therefore only draw large-scale conclusions about changes that have occurred over the study time period, based on patterns that are consistent over the majority of posterior draws. Second, this study compared genotype data generated using different approaches: data from 2013 to 2014 was obtained using MIPs and Illumina sequencing, while data from 2007 was obtained with standard PCR amplification and alternate sequencing methods. However, the sequencing coverage is approximately the same across studies, providing assurance that the methods are comparable. Additionally, the MIPs did not amplify across all of *pfdhps* in a single sequence but rather used multiple MIP probes to target the regions of interest. Therefore, we could not create true haplotypes across *pfdhps*.

## Conclusion

The findings from this study indicate that the prevalence and geographic spread of SP resistance increased between 2007 and 2013. In contrast, the proportion and pattern of chloroquine resistance stayed the same, potentially a result of ASAQ use or informal chloroquine use. These findings indicate a need to continue monitoring these resistant mutations to prevent additional spread, and to further investigate the factors driving these patterns.

## Supplementary information

**Additional file 1.** Additional description of the modeling methods used in the study as well as additional tables and figures to support our findings.

## Data Availability

Sequencing data used in this study are available in the NCBI SRA (Accession number PRJNA545347). Data used in this study from the Demographic Health Surveys Program are available upon request at https://dhsprogram.com/ [25]. Previously published data included in this study are available from the corresponding author upon reasonable request.

## Abbreviations

ASAQ: Artesunate/Amodiaquine
COI: Complexity of infection
DHS: Demographic and Health Surveys
DRC: Democratic Republic of the Congo
IPTp: Intermittent Preventative Therapy for Pregnant Women
MIP: Molecular inversion probe
PCR: Polymerase chain reaction
RFF: Random Fourier features
SNP: Single Nucleotide Polymorphism
SP: Sulfadoxine/pyrimethamine
