# Supplementary material for: The changing landscape of Plasmodium falciparum drug resistance in the Democratic Republic of Congo
Source: BMC Infect Dis. 2019 Oct 22;19:872. doi: 10.1186/s12879-019-4523-0 (PMC6805465; doi:10.1186/s12879-019-4523-0)
Supplement: Supplementary file 1 — Additional file 1. Additional description of the modeling methods used in the study as well as additional tables and figures to support our findings. [file 12879_2019_4523_MOESM1_ESM.docx]

**Additional file 1**

**Text S1**

**The changing landscape of *Plasmodium falciparum* drug resistance in the Democratic Republic of Congo**

**Molly Deutsch-Feldman^1*^, Ozkan Aydemir^2^, Margaret Carrel^3^, Nicholas F. Brazeau^1^, Samir Bhatt^4^, Jeffrey A. Bailey^2^, Melchior Kashamuka^5^, Antoinette K. Tshefu^5^, Steve M. Taylor^6^, Jonathan J. Juliano^1,7,8^, Steven R. Meshnick^1^, and Robert Verity^4^.**

1. Department of Epidemiology, Gillings School of Global Public Health, University of North Carolina, Chapel Hill, USA

2. Department of Pathology and Laboratory Medicine, Brown University, Providence, Rhode Island, USA

3. Department of Geographical & Sustainability Sciences, University of Iowa, Iowa City, IA, USA

4. Medical Research Council Centre for Global Infectious Disease Analysis, Department of Infectious Disease Epidemiology, Imperial College London, UK

5. Kinshasa School of Public Health, Hôpital General Provincial de Reference de Kinshasa, Kinshasa, Democratic Republic of Congo

6. Division of Infectious Diseases and Duke Global Health Institute, Duke University, Durham, North Carolina, USA

7. Division of Infectious Diseases, University of North Carolina at Chapel Hill, USA

8. Curriculum in Genetics and Molecular Biology, University of North Carolina at Chapel Hill, USA

*corresponding author: [mollydf1@live.unc.edu](mailto:mollydf1@live.unc.edu), 301-675-4599

*Epidemiologic Risk Factor Model Specification:*

The mixed-effect epidemiologic risk factor models were of the following form:

$$\Pr\left( Y_{hi} \right)=\beta_{0}+\beta X_{hi}+\mu_{0h}+\epsilon_{hi}$$

$\mu_{0h} \sim N(0, \sigma_{\mu0}^{2}$)

$$\epsilon_{hi} \sim N(0, \sigma_{e0}^{2})$$

Here, Y is a vector of response variables for each individual i within each province h, X is a vector of risk factor covariates. Mu is a random effect for each province and epsilon is an error term for each individual. For the multivariate model, the initial model was fit with all potential risk factors. The univariate models were each individually fit with only a single risk factor.

*Spatial-temporal Model Specification:*

The spatial-temporal model was built around the logistic Gaussian process ^1^, in which a smoothly varying$d$-dimensional Gaussian process is transformed to produce values constrained to the $[0,1]$ interval. These transformed values were then used to represent underlying frequencies of drug resistant alleles, and the observed counts were modelled as Binomial draws from these frequencies. The Gaussian process can be defined in any number of dimensions, giving the flexibility to model any number of predictor variables – in our case space (in two-dimensions), time, and three additional covariates: accessibility ^2^, night time lights ^3^ (as a measure of population density) and proportion urban/rural ^4^, for a total of $d$ = 6 dimensions. Each dimension was given a separate weight using the vector $\gamma$ of scale parameters, and an overall scale parameter $\tau$ was applied over all dimensions. Independent $Gamma(1,1)$ priors were applied on the$\gamma$ parameters in each dimension, and an${Gamma}^{-1}\left( 1,1 \right)$ prior was applied on the overall scale parameter $\tau$.

To facilitate model fitting, the complete logistic Gaussian process model described above was replaced by a lower-dimensional approximation using the method of random Fourier features^5^ (RFF). In simple terms, RFF can be understood as a way of decomposing a complex function into the sum over a number of “features”, at which point the model becomes a simple linear model over these features. The number of features used in the RFF approximation can be chosen by the user based on the relative merits of computational simplicity (for a small number of features), to fidelity to the true Gaussian process (for a large number of features). In our case, we found that 250 features captured the model well and ran in acceptable time. More specifically, the RFF method uses a feature map that associates a kernel function $k:\mathcal{X\times X\longrightarrow}\mathbb{R}$ which is defined on an input domain $\mathcal{X\in}\left\{ x_{1},..,x_{d} \right\}\in\mathbb{R}^{d}$such that the kernel between points $x_{i}$ and $x_{j}$, $k\left( x_{i},x_{j} \right)$ can be written $k\left( x_{i},x_{j} \right)=\left\langle\phi\left( x_{i} \right),\phi\left( x_{j} \right) \right\rangle_{\mathcal{H}}$ where $\phi:\mathcal{X\longrightarrow H}$ is the feature map that associates kernel $k$ with an embedding of the input space into a reproducing kernel Hilbert space $\mathcal{H}$. Following from Rahimi et al 2008^5^ our feature map takes the form $\phi=\left[ \cos\left( x^{T}\omega\right)\sin\left( x^{T}\omega\right) \right]^{T}$ such that $k\left( x_{i},x_{j} | \theta\right)\approx\frac{\sigma^{2}}{N_{feat}}\cdot\sum_{r=1}^{M} \phi^{T}\phi$, with given fixed spectral measure $\omega$. We assume a Gaussian spectral distribution corresponding to a squared exponential kernel. The final model can be written:

$y\sim Binomial\left( \sigma\left( \beta\phi^{T} \right)^{-1} \right)$

$$\phi\sim\left[ \cos\left( {\gamma\circ x}^{T}\omega\right)\sin\left( {\gamma\circ x}^{T}\omega\right) \right]^{T}$$

$$\beta\sim MVN(0,\tau I)$$

$$\tau\sim Gamma^{-1}(1,1)$$

$$\gamma\sim Gamma(1,1)$$

$$\omega=MVN(0,I_{D})$$

Where $y$ are the observed binary response variables, $\sigma$ is the logit transform, and hence $\sigma^{-1}$ is the logistic transform. This model was fitted using Hamiltonian Monte Carlo using the R package Greta^6,7^ using 10,000 burn-in iterations, 1 million sampling iterations, and thinning to ever 100^th^ value to remove autocorrelation.

**Table S1**: **Results from the univariate risk factor analyses.** Univariate mixed-effects model results for any *pfdhps* mutation, *pfdhps* K540E (including those with the A581G mutation also), and the *pfcrt* CVIET haplotype. Associations with a p-value <0.05 are bolded.

|  | **Any *pfdhps* mutation** | | ***Pfdhps* K540E** | | ***Pfcrt* CVIET haplotype** | |
| --- | --- | --- | --- | --- | --- | --- |
| Covariate | Prevalence  ratio (95% CI) | p-value | Prevalence ratio (95% CI) | p-value | Prevalence ratio (95% CI) | p-value |
| 10% Prevalence increase | 1.00  (0.95 - 1.05) | 0.92 | 1.09  (1.00 – 1.20) | 0.06 | 0.99  (0.94 – 1.04) | 0.66 |
| 10% Drug use increase* | 1.00  (0.95 - 1.06) | 0.89 | 1.13  (1.03 – 1.23) | **< 0.01** | 1.01  (1.00 – 1.02) | 0.48 |
| Individual wealth index | 0.96  (0.89 - 1.04) | 0.32 | 1.04  (0.90 – 1.19) | 0.59 | 1.08  (0.99 – 1.18) | 0.10 |
| 10% increase in lowest wealth category | 0.99  (0.96 - 1.05) | 0.98 | 0.94  (0.87 – 1.02) | 0.13 | 0.95  (0.90 – 1.00) | 0.07 |
| 10% increase in lowest education category | 1.02  (0.97 - 1.07) | 0.45 | 1.00  (0.93 – 1.09) | 0.90 | 0.92  (0.87 – 0.98) | **<0.01** |
| Urban | 0.96  (0.77 - 1.20) | 0.74 | 1.48  (0.86 - 2.57) | 0.16 | 1.21  (0.95 - 1.54) | 0.12 |
| Cluster size | 1.01  (0.99 - 1.01) | 0.72 | 0.99  (0.98 - 1.01) | 0.38 | 1.00  (1.00 - 1.01) | 0.37 |
| Female Sex | 1.02  (0.92- 1.14) | 1.12 | (0.95 – 1.32) | 0.48 | 1.06  (0.95 – 1.17) | 0.593 |

*SP amongst pregnant women for *pfdhps* models and chloroquine amongst children for the *pfcrt* model

**Figure S1:** Posterior 95% credible intervals on weights for all covariates included in spatial-temporal prediction model for A) A437G B) K540E C) A581G and D) CVIET


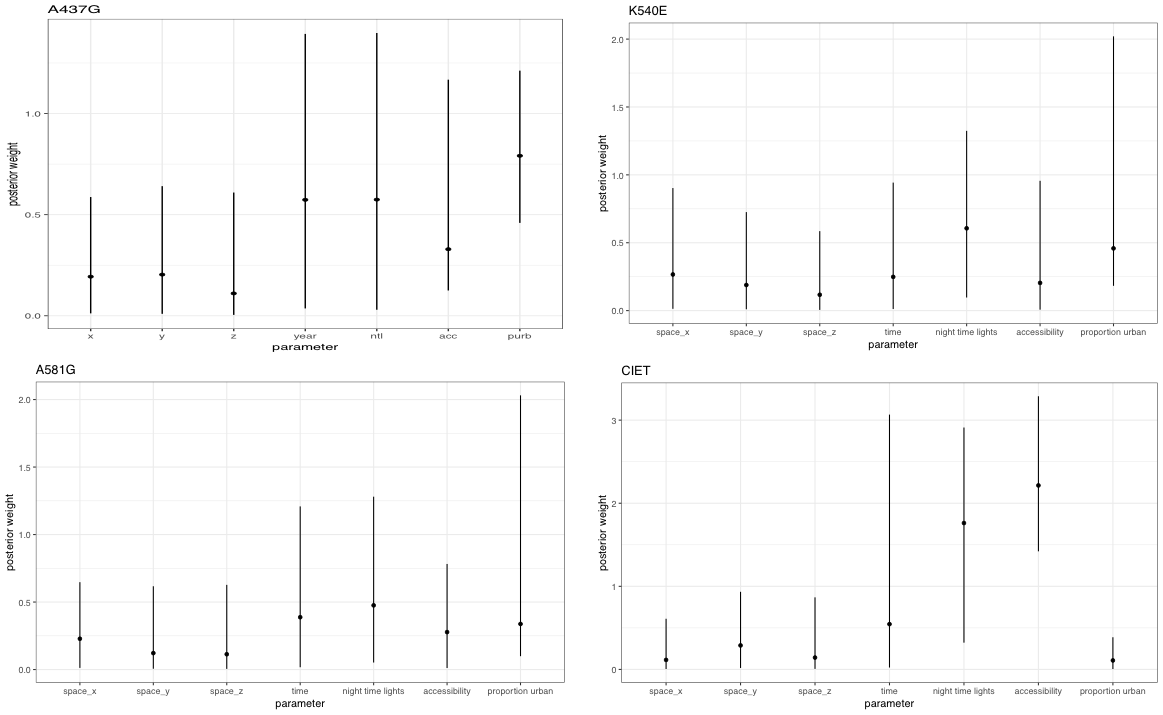


**Figure S2: Mean posterior standard deviation of estimated allele frequencies for each mutation**

References

1. Rasmussen, Carl Edward (2004). "Gaussian processes in machine learning." *Advanced lectures on machine learning*. Springer, Berlin, Heidelberg. 63-71.

2. Weiss, D. J., et al. (2018). "A global map of travel time to cities to assess inequalities in accessibility in 2015." *Nature* 553.7688: 333.

3. Noor, Abdisalan M., et al. (2008). "Using remotely sensed night-time light as a proxy for poverty in Africa." *Population Health Metrics* 6.1: 5.

4. Esch, Thomas, et al. (2017). "Breaking new ground in mapping human settlements from space–The Global Urban Footprint." *ISPRS Journal of Photogrammetry and Remote Sensing* 134: 30-42.

5. Rahimi, Ali, and Benjamin Recht (2008). "Random features for large-scale kernel machines." *Advances in neural information processing systems*.

6. R Core Team (2018). “R: A Language and Environment for Statistical Computing.” *R Foundation for Statistical Computing, Vienna, Austria.*

7. Golding, Nick (2018). "greta: Simple and Scalable Statistical Modelling in R." *URL: https://cran. r*.
